# Preoperative geriatric nutritional risk index is an independent prognostic factor for postoperative survival after gallbladder cancer radical surgery

**DOI:** 10.1186/s12893-022-01575-2

**Published:** 2022-04-07

**Authors:** Huifan Dai, Jing Xu

**Affiliations:** Department of Endocrinology, The Second Affiliated Hospital and Yuying Children’s Hospital of Wenzhou Medical University, Lucheng District, Wenzhou, Zhejiang Province People’s Republic of China

**Keywords:** Geriatric nutritional risk index, Gallbladder cancer, Survival, Nutritional, Prognosis

## Abstract

**Background:**

Currently, the surgical outcomes of gallbladder cancer (GBC) are not always satisfactory. The geriatric nutritional risk index (GNRI) can effectively assess nutritional status. This study intends to investigate whether the preoperative GNRI can predict the prognosis of GBC.

**Methods:**

202 consecutive GBC patients who underwent treatment from 2010 to 2017 were selected and analyzed retrospectively. By using the univariate and multivariate Cox regression analyses on overall survival (OS) and recurrence-free survival (RFS), the preoperative GNRI of GBC patients was evaluated.

**Results:**

Among the 202 patients, the GNRI of the 86 patients (42.6%) was less than 98. The patients with low preoperative GNRI had the median OS of 26 months, which was less than the median OS of 39 months among those patients who had higher preoperative GNRI (P < 0.001). Univariate analysis showed that low GNRI was related to short survival time (HR 3.656, 95% CI 2.308–5.790, P < 0.001). In addition, the results of multivariate analysis revealed that, the patients with low GNRI showed a lower OS (HR 2.207, 95% CI 1.131–4.308, P = 0.020) and RFS (HR 2.964, 95% CI 1.577–5.571, P = 0.001) than those patients with higher GNRI.

**Conclusion:**

GNRI is an independent indicator of poor prognosis in GBC patients after GBC radical surgery.

## Introduction

Gallbladder cancer (GBC) has become the most common malignancy of biliary tract tumor, accounting for 80–95% of biliary cancer patients [1]. GBC is the fifth most common gastrointestinal tract malignancy, accounting for about 1% of all cancers in China [2]. Owing to the insufficient effective diagnostic markers, and insufficient available treatment option, most patients were diagnosed as advanced stage, with atypical symptoms and poor prognosis [3]. In general, the prognosis of GBC has been primarily determined and identified by tumor pathology stage, pathologic type and tumor classification. The results of epidemiologic studies showed that patients diagnosed with this disease had 30% 3-years survival rates and 5% 5-years survival rates, respectively [4]. Therefore, considering the ineffective prognosis for GBC, it is necessary to find other factors that mayaffect the survival rate of GBC patients in further research.

The association between survival rate of patients with organ malignancies (e.g., gallbladder cancer) and their nutritional status is a research hotspot in recent years. Some researchers have demonstrated that there are associations between preoperative nutritional status and postoperative complications, as well as long-term prognosis results of malignant tumors patients [5–7]. As we all know, the serum albumin concentration is considered as an index of protein reserves. In recent years, more and more researches have confirmed that low serum albumin are critical poor prognosis predictors in many cancers. In contrast, body mass index (BMI) is regarded as a reasonable adiposity evaluation tool, the BMI exceeding normal range is a risk factor for death from cancer, stroke, and ischemic heart disease [8].

The geriatric nutritional risk index (GNRI) is an indicator of malnutrition, which can be used as an uncomplicated and important factor for predicting the results achieved from only serum albumin and the rate between ideal and practical body weights. GNRI has been analyzed in many studies recently, its prognostic value was demonstrated for patients on diffuse large B cell lymphoma, esophageal cancer, cardiovascular diseases and chronic liver failure [9–12]. However, whether the GNRI is feasible as the prognosis indicator of organ malignancies (e.g., GBC) is still unclear.

Accordingly, we hypothesized that the GNRI could be an applicable prognostic factor of GBC patients after radical surgery. This study aims to retrospectively investigate whether preoperative GNRI can be a prognostic indicator for survival rate of patients with GBC.

## Materials and methods

In this study, a retrospective analysis was carried out according to the Helsinki Declaration and approved by the Ethics Committee of the Second Affiliated Hospital of Wenzhou Medical University. The written informed consent of all patients was collected. Patients who met the inclusion criteria were involved in this study, which included (1) Patients diagnosed as GBC with histology. (2) GBC patients with no other coexisting malignancies. (3) Patients didn’t undergo other treatment methods before recruitment. (4) The follow-up data and overall clinical data of patients were available and credible. (5) Patients aged > 18 years old. While the exclusion criteria included (1) Patients suffered from acute infection or chronic active inflammatory disease. (2) Patients with anemia, collagen diseases and others diseases related to the hematological system. (3) Patients had been taking anticoagulant treatment or albumin transfusions before having the anti-tumor treatment. (4) Patients represented perioperative surgery-associated mortality. Subsequently, the clinical data within 30 days before surgery for 202 GBC patients were collected. In the Second Affiliated Hospital of Wenzhou Medical University, all patients underwent the GBC radical operation from 2010 to 2017. The researcher carried out the follow-up with telephone for the patients once a year to collect and record their recent physical indicators. The follow-up data included the data from surgery to the death date or the last contacting date, or the end of November 2019. The dates from the surgery to the last follow-up or death are defined as overall survival (OS). Recurrence free survival (RFS) was calculated according to the date of surgery to the first recurrence or death due to some causes, or the last follow-up date.

### Clinical information

The following medical data were retrospectively collected from the patients in the hospital, including TNM staging (The United States Joint Committee on cancer [7th edition], staging of gallbladder cancer), pathologic data (e.g., tumor classification, tumor size, tumor lymph node metastasis), serum carcinoembryonic antigen (CEA), serum CA19-9, neutrophil-to-lymphocyte ratio (NLR), serum albumin, hemoglobin, BMI, age, and gender. Based on electrochemiluminescence immunoassays (Cobas; Roche Diagnostics, Germany) performed in the Clinical Laboratory Department of the Second Affiliated Hospital of Wenzhou Medical University, serum CA19-9 and CEA levels were identified. The normal reference values in line with a previous study are as follows: CEA ≤ 5 μg/L, CA19-9 ≤ 37 U/mL, NLR < 2.6 [13]. The radical resection is defined that the primary tumor is removed together with the tissue invaded by cancer cells and the metastatic lymph nodes. (For patients with the T1 a GBC, we performed a simple cholecystectomy using a laparotomy or laparoscopic surgery. For patients with the T1b GBC, an extended cholecystectomy was employed. For patients with GBC in stage T2 or advanced level, an extended cholecystectomy was frequently performed. In the extended cholecystectomy, IVb/V liver resection or the gallbladder bed wedge resection could be conducted according to the intraoperative condition. The scope of lymph node dissection should include the posterior superior pancreaticoduodenal lymph node, the hepatic artery and portal vein lymph nodes, the lymph nodes around the hepatoduodenal ligament the common bile duct lymph node, as well as the cystic duct lymph node). According to the tumor pathological diagnosis, there was no tumor tissue at the margin.

### GNRI calculation

GNRI was calculated according to individual serum albumin levels, ideal body weight (kg), actual body weight (kg), and height (cm), as shown in the below function [14]:

$${\text{GNRI}} = \left( {{1}.{489}*{\text{albumin }}\left( {{{\text{g}} \mathord{\left/ {\vphantom {{\text{g}} {\text{L}}}} \right. \kern-\nulldelimiterspace} {\text{L}}}} \right)} \right) + \left( {{41}.{7}*\left( {{{{\text{weight}}} \mathord{\left/ {\vphantom {{{\text{weight}}} {{\text{WL}}0}}} \right. \kern-\nulldelimiterspace} {{\text{WL}}0}}} \right)} \right),$$

Body weight/WL0 was set as 1 when the body weight of patients exceeded the ideal body weight.

WL0 refers to the ideal weight, which is calculated by the following function:

$${\text{Male}}:{\text{WL}}0 = {\text{H}} - {1}00 - \left( {\left( {{\text{H}} - {15}0} \right)/{4}} \right)$$

$${\text{Female}}:{\text{WL}}0 = {\text{H}} - {1}00 - \left( {\left( {{\text{H}} - {15}0} \right)/{2}.{5}} \right)$$

H refers to the height.

Based on the calculated values of GNRI, two grades of risk related to nutrition were defined: High risk (< 98), and low risk (≥ 98) [15].

### Statistical method

For categorical variables, significant differences were assessed by adopting the chi-square test or Fisher’s exact test. For continuous data, the mean diversification was compared by adopting independent-sample t-test or the Mann–Whitney test. In order to find the associations between OS and parameters, univariate and multivariate Cox proportional hazard models were applied in this study. Multivariate analyses were performed using the factors that were significant in univariate analyses. OS was defined as the time from the data of surgery to the date of the last follow-up or death from any cause. With the utilization of the Kaplan–Meier approach, the survival curves were explored. By conducting the log-rank test, the relevant comparison was drawn. The P-value < 0.05 was identified with the statistical difference. Statistical analyses were conducted with the SPSS version 22 (SPSS, Lnc., Chicago, IL, USA).

## Results

### Baseline characteristics

The 202 GBC patients were enrolled in the study, and the mean GNRI was 100.08 ± 10.69, among the GBC patients, 68.3% of them were female, and the mean age was 68.54 ± 11.02 years old. Besides, 42.6% GBC patients had low GNRI according to medical record. The fundamental characteristics of survivor and non-survivor patients are listed in Table 1. Compared with surviving patients, non-surviving patients were older, with lower GNRI, albumin, hemoglobin and BMI. In addition, high CA19-9 (P < 0.001) and high CEA (P = 0.006) between the survivor and non-survivor patients were considerably divergent. For tumor characteristics, advanced infiltration depth T (P < 0.001), advanced TNM stage (P < 0.001), tumor differentiation (P < 0.001) and lymph node metastasis (P = 0.046) considerably impacted the death of GBC patients. In addition, according to the GNRI value, the patients were divided into high GNRI groups (n = 116) and low GNRI groups (n = 86) (Table 2). In contrast with high GNRI patients, the low GNRI patients was older, with lower albumin, hemoglobin, BMI. And higher NLR (P < 0.001), CA19-9 (P = 0.017), incidence of gallstones (P = 0.012) and poor tumor differentiation (P = 0.042) had significant effects in patients with low GNRI level.

**Table 1:** The comparison of the clinical and pathologic between survivors and non-survivor groups

	Overall	Survivors	Non-survivors	P value
Case n (%)	202	108	94	
Age, years	68.54 ± 11.02	66.81 ± 10.87	70.52 ± 13.91	0.017
Female (%)	68.3%	70.4%	66.0%	0.546
Height, cm	159.56 ± 7.44	159.39 ± 7.69	159.76 ± 7.16	0.728
Weight, kg	58.31 ± 9.87	56.42 ± 8.96	59.96 ± 10.36	0.011
BMI, kg/m^2^	22.87 ± 3.29	23.58 ± 3.53	22.05 ± 2.80	0.001
GNRI	100.08 ± 10.69	104.12 ± 9.80	95.44 ± 9.78	< 0.001
Hemoglobin mean, g/L	124.94 ± 17.97	127.12 ± 14.43	122.43 ± 21.13	0.044
Serum albumin mean, g/L	38.30 ± 5.18	40.10 ± 4.19	36.23 ± 5.44	< 0.001
NLR				0.112
< 2.6	61.4%	66.7%	55.3%	
≥ 2.6	38.6%	33.3%	44.7%	
Serum CA19-9 (U/mL) > 37				< 0.001
No	58.9%	72.2%	43.6%	
Yes	41.1%	27.8%	56.4%	
Serum CEA level (ng/mL) > 5				0.006
No	77.5%	86.0%	67.5%	
Yes	22.5%	14.0%	32.5%	
T (%)				< 0.001
I–II	34.9%	48.0%	20.9%	
III–IV	65.1%	52.0%	79.1%	
Gallstones (%)				0.077
No	39.9%	46.1%	33.0%	
Yes	60.1%	53.9%	67.0%	
Tumor size (cm) > 3 (%)				1.000
No	53.2%	53.6%	52.9%	
Yes	46.8%	46.4%	47.1%	
Differentiation of GBC (%)				< 0.001
Poor/unknown	37.4%	24.2%	52.5%	
Well/moderate	62.6%	75.8%	47.5%	
TNM stage (%)				< 0.001
I–II	30.5%	46.2%	12.8%	
III–IV	69;.5	53.8%	87.2%	
Lymph node metastases (%)				0.046
No	75.3%	80.8%	69.2%	
Yes	24.7%	19.2%	30.8%	
GNRI				< 0.001
≥ 98	57.4%	69.8%	30.2%	
< 98	42.6%	31.4%	68.6%

**Table 2:** Clinical and pathologic characteristics of the 202 study patients

	Overall	High GNRI (≥ 98)	Low GNRI (< 98)	P value
Case n (%)	202	116 (57.4%)	86 (42.6%)	
Age, years	68.54 ± 11.02	66.51 ± 9.89	71.28 ± 11.90	0.001
Female (%)	68.3%	73.3%	61.6%	0.093
BMI, kg/m^2^	22.87 ± 3.29	24.23 ± 3.05	21.03 ± 2.66	< 0.001
Hemoglobin mean, g/L	124.94 ± 17.97	129.18 ± 16.62	119.21 ± 18.22	< 0.001
Serum albumin mean, g/L	38.30 ± 5.18	41.44 ± 3.11	34.07 ± 4.32	< 0.001
NLR				< 0.001
< 2.6	61.4%	75.0%	43.0%	
≥ 2.6	38.6%	25.0%	57.0%	
Serum CA19-9 (U/mL) > 37				0.017
No	58.9%	67.0%	47.9%	
Yes	41.1%	33.0%	52.1%	
Serum CEA level (ng/mL) > 5				0.145
No	77.5%	81.4%	71.8%	
Yes	22.5%	18.6%	28.2%	
T (%)				0.280
I–II	34.9%	38.5%	30.0%	
III–IV	65.1%	61.5%	70.0%	
Gallstones (%)				0.012
No	39.9%	47.7%	29.8%	
Yes	60.1%	52.3%	70.2%	
Tumor size (cm) > 3 (%)				0.408
No	53.2%	56.4%	48.3%	
Yes	46.8%	43.6%	51.7%	
Differentiation of GBC (%)				0.042
Poor/unknown	37.4%	32.0%	45.1%	
Well/moderate	62.6%	68.0%	54.9%	
TNM stage (%)				0.354
I–II	30.5%	33.3%	26.7%	
III–IV	69;.5	66.7%	73.3%	
Lymph node metastases (%)				0.610
No	75.3%	77.1%	72.8%	
Yes	24.7%	22.9%	27.2%	

### Kaplan–Meier survival analysis

**Fig. 1:**
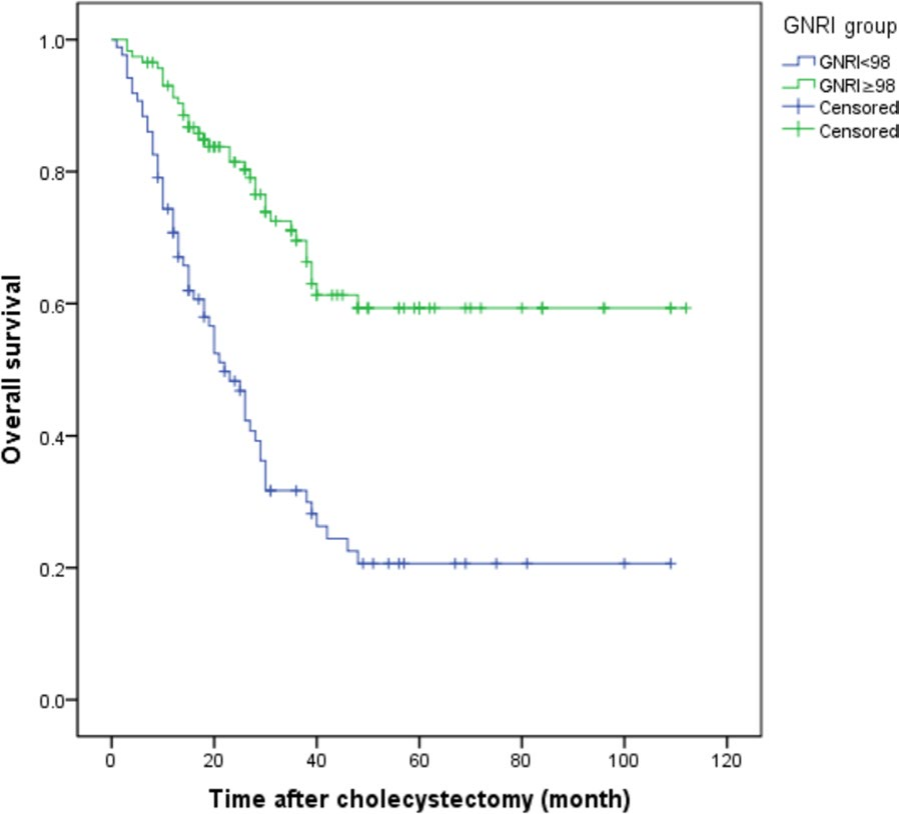
The overall survival rate of GBC patients after GBC radical surgery. *Notes* The Kaplan–Meier curve showed significant differences in the probability of total survival after GBC radical surgery in patients with GNRI < 100 and GNRI ≥ 100. P < 0.001 (log-rank test)

**Fig. 2:**
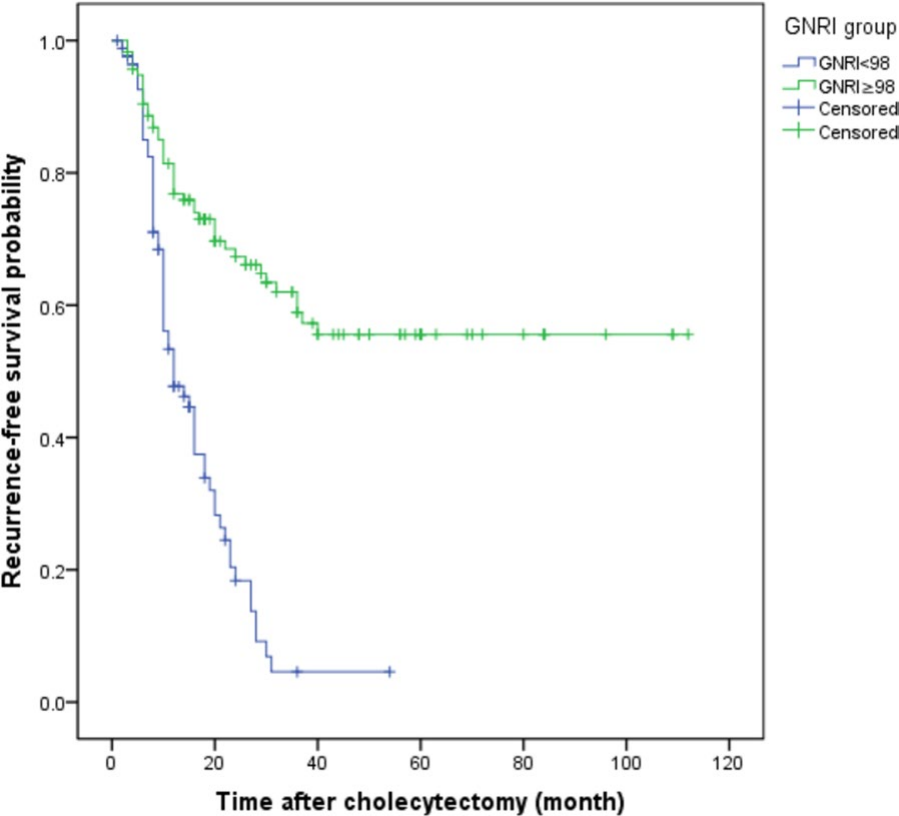
RFS of patients with GBC after GBC radical surgery. *Notes* Kaplan–Meier curves show significant difference in RFS probability after GBC radical surgery in patients with preoperative high GNRI and low GNRI levels. P < 0.001 (log-rank test)

Among the 202 patients, 94 patients died and 108 patients survived. For the OS and RFS (P < 0.001) (Figs. 1, 2), the prognosis was considerably worse in patients with poor GNRI levels than in patients with high GNRI levels. Patients in the high GNRI level group showed a medium OS time of 39 months (95% CI 29.78–48.22), while the patients with the low GNRI showed a mean OS of 22 months (95% CI 16.61–27.39). The recurrence rate during following-up reached 52.0% (105 cases), and the middle illness-free survival reached 24 months. The middle RFS significantly shorter in the low GNRI group than that in the high GNRI group (12 months and 35 months, separately, P < 0.01).

### Univariate and multivariate analysis

The OS-related variables after GBC radical surgery complying with univariate and multivariate Cox proportional hazard models are listed in Table 3. In the univariate analysis, older age, serum CA19-9 > 37, CEA > 5, hemoglobin level, invasive depth III + IV, poor differentiation, lymph node metastasis and GNRI < 98 were associated with low OS. According to the results of multivariate analysis, age (HR 1.030, 95% CI 1.002–1.060, P = 0.038), hemoglobin (HR 1.023, 95% CI 1.001–1.047, P = 0.047), poor differentiation (HR 2.050, 95% CI 1.070–4.300, P = 0.042), TNM III + IV (HR 8.035, 95%CI 1.708–37.812, P = 0.008) and low GNRI (HR 2.207, 95% CI 1.131–4.308, P = 0.020) adversely affected OS. Table 4 lists a multiple-variate and univariate Cox proportional hazard regression mode for RFS. By multivariate analysis, this study identified two adverse prognosis factors affecting RFS, namely, TNM stage (HR 2.308, 95% CI 1.051–7.567, P = 0.047) and GNRI (HR 2.964, 95% CI 1.577–5.571, P = 0.001).

**Table 3:** Univariate and multivariate analyses of prognostic factors with overall survival

Variables	Univariate analysis		Multivariate analysis	
	HR (95%CI)	P-value	HR (95% CI)	P-value
Age	1.026 (1.006–1.046)	0.009	1.030 (1.002–1.060)	0.038
Sex male	1.195 (0.779–1.832)	0.414		
serum CA 19-9 (U/mL) > 37	2.304 (1.470–3.612)	< 0.001	1.633(0.822–3.244)	0.161
Serum CEA (ng/mL) > 5	1.904 (1.192–3.041)	0.007	1.674(0.815–3.441)	0.161
Hemoglobin	1.192 (1.098–1.214)	0.038	1.023 (1.001–1.047)	0.047
NLR > 2.6	1.447 (0.963–2.174)	0.075		
Gallstones	1.271 (0.821–1.970)	0.282		
Tumor size > 3 cm	1.021 (0.639–1.633)	0.930		
T III–IV	1.630 (1.253–2.121)	< 0.001	0.338 (0.098–1.160)	0.085
Poor differentiation	2.323 (1.496–3.607)	< 0.001	2.050 (1.070–4.300)	0.042
Lymph node metastases	1.567 (1.004–2.448)	0.048	0.806 (0.388–1.674)	0.563
TNM stage III + IV	3.688 (2.011–6.764)	< 0.001	8.035 (1.708–37.812)	0.008
GNRI < 98	3.194 (2.098–4.861)	< 0.001	2.207 (1.131–4.308)	0.020

**Table 4:** Univariate and multivariate analyses of prognostic factors associated with RFS

Variables	Univariate analysis		Multivariate analysis	
	HR (95%CI)	P-value	HR (95% CI)	P-value
Age	1.016(0.998–1.034)	0.074		
Sex male	0.808(0.539–1.210)	0.301		
serum CA 19-9 (U/mL) > 37	1.928(1.263–2.945)	0.002	1.403 (0.742–2.655)	0.298
Serum CEA (ng/mL) > 5	1.631(1.025–2.593)	0.039	1.175 (0.567–2.436)	0.664
Hemoglobin	0.994(0.982–1.006)	0.306		
NLR > 2.6	1.527(1.036–2.251)	0.032	1.239 (0.635–2.417)	0.530
Gallstones	1.506(0.995–2.279)	0.053		
Tumor size > 3 cm	0.892(0.570–1.396)	0.617		
T III–IV	1.112 (0.904–1.368)	0.314		
Poor differentiation	1.640(1.084–2.484)	0.019	1.779 (0.937–3.378)	0.078
Lymph node metastases	3.318 (1.128–7.048)	0.014	0.950 (0.481–1.879)	0.884
TNM stage III + IV	1.617 (1.037–2.520)	0.034	2.308 (1.051–7.567)	0.047
GNRI < 98	3.870 (2.560–5.851)	< 0.001	2.964 (1.577–5.571)	0.001

## Discussion

GBC has been considered as a rare disease and the most common incursive tumor in the biliary system [1, 16]. Nevertheless, GBC progresses rapidly and rarely presents rare early symptom [17]. Accordingly, early detection and early surgical treatment are advocated [16], whereas GBC prognostic process is still not effective. Therefore, new and accurate prognosis biomarkers for GBC are required to investigate. We found that low GNRI was identified as an effective predicting factor of OS and RFS in GBC patients after they underwent GBC radical surgery.

In general, serum albumin level and BMI are the key variables that can reflect the survival risk of malignant tumor patients. Therefore, serum albumin is used as a highly correlated malnutrition index and a useful index of malnutrition or cachexia in cancer patients. As this condition is partially reflected by hypoalbuminemia, nutrition can be employed as a critical factor to determine immune response [18]; On the basis of this theory, the hypothesis is that hypoalbuminemia may show associations with damaged immune response. In addition, a systematic review shows that high serum albumin concentration is associated with higher survival outcomes of cancer patients [19].

Previous studies have demonstrated an association between obesity and risk of GBC incidence [20], which also has indicated that body weight loss refers to an ineffective prognosis factor of GBC. According to Kang et al., survival rate was considerably better in patients with large BMI compared with those with small BMI. Furthermore, body weight loss was expected under negative cell regulating mechanisms for cancer or in patients with aggressive cancer. Nevertheless, low BMI or body weight loss refers to a negative prognosis factor for GBC patients [21].

According to Kawai et al., nutrition critically determines immune responses, and malnutrition most frequently leads to immunodeficiency [22]. Furthermore, malnutrition is related to immune damage mediated by host cells resistant to cancer. Malnutrition is a state of protein-energy or other nutritional imbalance, as well as a feature of cachexia. Systemic inflammation in the course of tumor progression will result in cachexia in cancer patients. In cancer patients with cachexia, elevated circulating concentrations of a range of inflammatory cytokines were identified [23]. Given the above results, malnutrition is likely to confirm poor outcome even in GBC patients after resection surgery.

GNRI depends on weight loss and serum albumin concentration, revealing nutritional status. Accordingly, the GNRI can assess the above 2 variables in the meantime. In addition, GNRI can be simply calculated according to laboratory data acquired in routine laboratory examination, with the efficacy of assessing the nutritional status. Nevertheless, few studies have verified the clinics-based application of GNRI for organ malignancies [11].

In this study, approximate 42.6% of patients had GNRI less than 98.However, according to the results of preoperative functional test and appearance of these patients, they all seemed to be in a healthy enough state to undergo surgical resection. As revealed by the mentioned result, preoperative GNRI can be adopted to effectively to identifying patients undernourished among all patients with appropriate organ functions and allowed to receive surgery.

In this study, compared with patients with large GNRI before surgery, patients with poor GNRI exhibited larger NLR (P < 0.001), CA19-9 (P = 0.017), incidence of gallstones (P = 0.012) and poorer tumor differentiation (P = 0.042). Accordingly, patients with poor GNRI were likely to show tumor development. Therefore, such variable is likely to be adopted as an effective index of high-grade tumor malignancy. Nevertheless, we cannot draw the conclusion that low GNRI is likely to lead to or be attributed to tumor malignancy only according to the data in this study.

Furthermore, multivariate analysis showed that poor differentiation, low hemoglobin, high age, low GNRI, lymph node metastases and TNM III + IV were independent prognostic factors in GBC patients. Considering our knowledge, it was first reported in this study that a preoperative low GNRI is a predictive and prognostic factor of GBC. Moreover, it was reported that low GNRI patients had considerably shorter OS than high GNRI patients. Accordingly, GBC and low GNRI patients are at high-risk of death after undergoing therapeutic processes. Therefore, more careful follow-up should be conducted accordingly after undergoing surgeries in such kind of patients.

Bo et al. reported that the GNRI estimated survival in the elderly esophageal cancer patients with radiotherapy [11]. On the basis of the conclusion drawn by these researchers, the GNRI was an independent prognosis factor in elderly esophageal cancer patients underwent radiotherapy. Hayama et al. maintained that low-preoperative GNRI was significantly associated with a poor prognosis in elderly colorectal cancer patients [24]. Sakamoto fined that GNRI might be useful to predict prognosis in patients aged ≥ 65 years with pancreatic cancer [25]. Liu et al. found that GNRI maybe used to assess malnutrition in older adult cancer patients and can predict poor clinical outcomes in these patients [26], which has supported the findings of our study simultaneously.

However, there are several limits in this study. Firstly, it was a single-center study with a relatively small sample size, the results may not be representative for patients with GBC. Secondly, the GNRI values were not measured with a disease-specific scale, such as the GBC scale, which would provide a more adequate measurement. Despite these limitations, the primary data are presented, which has revealed that the GNRI is a vital prognosis factor in GBC patients. Since the GNRI is an easily available clinical parameter, which can be considered as a very effective and potential index in standard clinical application.

## Conclusions

In conclusion, the preoperative GNRI refers to a new predicting factor and prognosis factor, and it has the potential value to be applied to determine which GBC patients should undergo multimodality therapies. In the further study, a prospective study is expected to be conducted to assess the survival benefit of multimodality therapies for GBC patients with low GNRI.

## Data Availability

The data that support the findings of this study are available from Institutional Review Board of the second affiliated hospital and Yuying Children’s Hospital of Wenzhou Medical University but restrictions apply to the availability of these data, which were used under license for the current study, and so are not publicly available. Data are however available from the authors upon reasonable request and with permission of Institutional Review Board of the Second Affiliated Hospital and Yuying Children’s Hospital of Wenzhou Medical University.

## Abbreviations

GNRI: Geriatric nutritional risk index
GBC: Gallbladder cancer
OS: Overall survival
CEA: Carcinoembryonic antigen
NLR: Neutrophil-to-lymphocyte ratio
