# Correction to: Preoperative geriatric nutritional risk index is an independent prognostic factor for postoperative survival after gallbladder cancer radical surgery

**DOI:** 10.1186/s12893-022-01731-8

**Published:** 2022-08-04

**Authors:** Huifang Dai, Jing Xu

**Affiliations:** Department of Endocrinology, The Second Affiliated Hospital and Yuying Children’s Hospital of Wenzhou Medical University, Lucheng District, Wenzhou, Zhejiang Province People’s Republic of China

## Correction to: BMC Surgery (2022) 22:133 https://doi.org/10.1186/s12893-022-01575-2

Following the publication of the original article [1], the author name ‘Huifang Dai’ has been misspelled as ‘Huifan Dai’.

The original article has been corrected.
